# Assessing ChatGPT’s Educational Potential in Lung Cancer Radiotherapy From Clinician and Patient Perspectives: Content Quality and Readability Analysis

**DOI:** 10.2196/69783

**Published:** 2025-08-13

**Authors:** Cedric Richlitzki, Sina Mansoorian, Lukas Käsmann, Mircea Gabriel Stoleriu, Julia Kovacs, Wulf Sienel, Diego Kauffmann-Guerrero, Thomas Duell, Nina Sophie Schmidt-Hegemann, Claus Belka, Stefanie Corradini, Chukwuka Eze

**Affiliations:** 1 Department of Radiation Oncology, University Hospital LMU, Marchioninistrasse 15, Munich, 81377, Germany, 49 89440073770; 2 Asklepios Lung Clinic Munich - Gauting, Division of Thoracic Surgery, LMU University Hospital, Munich, Germany; 3 Division for Thoracic Surgery, Asklepios Medical Center, Ludwig-Maximilians-University of Munich, Munich, Germany; 4 Department of Medicine V, University Hospital, Ludwig-Maximilians-University Munich, Munich, Germany; 5 Comprehensive Pneumology Center Munich, German Center for Lung Research, Munich, Germany; 6 German Cancer Research Center (DKFZ), German Cancer Consortium (DKTK), partner site Munich, Heidelberg, Germany; 7 Bavarian Cancer Research Center (BZKF), Munich, Germany

**Keywords:** artificial intelligence, LLM, large language model, patient education, ChatGPT, lung cancer, NSCLC, non-small cell lung cancer, radiotherapy, radiation oncology

## Abstract

**Background:**

Large language models (LLMs) such as ChatGPT (OpenAI) are increasingly discussed as potential tools for patient education in health care. In radiation oncology, where patients are often confronted with complex medical terminology and complex treatment plans, LLMs may support patient understanding and promote more active participation in care. However, the readability, accuracy, completeness, and overall acceptance of LLM-generated medical content remain underexplored.

**Objective:**

This study aims to evaluate the potential of ChatGPT-4 as a supplementary tool for patient education in the context of lung cancer radiotherapy by assessing the readability, content quality, and perceived usefulness of artificial intelligence–generated responses from both clinician and patient perspectives.

**Methods:**

A total of 8 frequently asked questions about radiotherapy for lung cancer were developed based on clinical experience from a team of clinicians specialized in lung cancer treatment at a university hospital. The questions were submitted individually to ChatGPT-4o (version as of July 2024) using the prompt: “I am a lung cancer patient looking for answers to the following questions.” Responses were evaluated using three approaches: (1) a readability analysis applying the Modified Flesch Reading Ease (FRE) formula for German and the 4th Vienna Formula (WSTF); (2) a multicenter expert evaluation by 6 multidisciplinary clinicians (radiation oncologists, medical oncologists, and thoracic surgeons) specialized in lung cancer treatment using a 5-point Likert scale to assess relevance, correctness, and completeness; and (3) a patient evaluation during the first follow-up appointment after radiotherapy, assessing comprehensibility, accuracy, relevance, trustworthiness, and willingness to use ChatGPT for future medical questions.

**Results:**

Readability analysis classified most responses as “very difficult to read” (university level) or “difficult to read” (upper secondary school), likely due to the use of medical language and long sentence structures. Clinician assessments yielded high scores for relevance (mean 4.5, SD 0.52) and correctness (mean 4.3, SD 0.65), but completeness received slightly lower ratings (mean 3.9, SD 0.59). A total of 30 patients rated the responses positively for clarity (mean 4.4, SD 0.61) and relevance (mean 4.3, SD 0.64), but lower for trustworthiness (mean 3.8, SD 0.68) and usability (mean 3.7, SD 0.73). No harmful misinformation was identified in the responses.

**Conclusions:**

ChatGPT-4 shows promise as a supplementary tool for patient education in radiation oncology. While patients and clinicians appreciated the clarity and relevance of the information, limitations in completeness, trust, and readability highlight the need for clinician oversight and further optimization of LLM-generated content. Future developments should focus on improving accessibility, integrating real-time readability adaptation, and establishing standardized evaluation frameworks to ensure safe and effective clinical use.

## Introduction

Artificial intelligence (AI) has made remarkable progress in recent years, with models like ChatGPT, launched by OpenAI in November 2022, emerging as critical tools in natural language processing. Built on the GPT architecture, ChatGPT has evolved from GPT-1 (2018) to GPT-4o (May 2024), with each iteration improving accuracy, contextual understanding, and versatility, particularly in generating human-like texts. In addition to ChatGPT, other notable large language models (LLMs) include Google’s Bard, which excels in generating creative content and integrating real-time data, Meta’s LLAMA, tailored for research and noncommercial applications, and Anthropic’s Claude, which prioritizes safety and ethical AI interactions.

ChatGPT, in its current form, offers notable advantages in the medical field, especially in patient education and communication [1,2]. It can provide clear explanations of complex medical concepts, answer patient queries, and assist clinicians in creating educational materials [3,4]. ChatGPT is a powerful tool for enhancing patient understanding and engagement in treatment plans, leveraging its ability to process and generate text from vast datasets.

In health care, ChatGPT has found diverse applications [5]. It is particularly effective for patient education, simplifying complex medical jargon into accessible language and offering support beyond clinical hours. Patients often have follow-up questions about treatment processes, side effects, safety, and treatment design and delivery [6].

These queries can significantly increase staff workload, potentially exacerbating physician burnout and negatively affecting care quality [7]. LLM chatbots like ChatGPT offer a promising solution to mitigate this burden by answering routine patient inquiries and reducing the workload on health care professionals. Furthermore, its ability to simulate conversations enables interactive patient education, improving comprehension and fostering a more informed and empowered patient community [4,8,9].

However, ChatGPT has limitations. It lacks critical thinking and contextual judgment, which can lead to misinformation or factually inaccurate responses, commonly referred to as “hallucinations” [10,11]. Other concerns include the risk of providing incorrect medical information, breaches of patient privacy, generating falsified documents or images, overdependence on AI in medical education, and reinforcing existing biases [12]. In addition, the current version relies on data up to 2021, limiting its ability to provide up-to-date medical information [13].

Lung cancer, one of the most prevalent cancers and the leading cause of cancer-related deaths worldwide, presents a significant need for patient education [14]. Given the critical implications of this diagnosis with its complex treatment modalities, such as radiotherapy, patients often seek comprehensive, reliable, and easily accessible information online [15].

This study aims to evaluate the educational capabilities and limitations of GPT-4 for patients undergoing radiotherapy for lung cancer. This includes a clinician-led evaluation of response relevance, accuracy, and completeness and a patient-led review of its performance in providing educational content. In addition, a readability analysis was conducted to assess the accessibility of the responses generated.

## Methods

### Overview

We, a team from the Department of Radiation Oncology at University Hospital Ludwig-Maximilians-University, Munich, Germany, designed 8 questions about radiotherapy for lung cancer. These questions were developed by collecting input from clinicians primarily involved in lung cancer treatment at our department, asking them which questions they frequently encounter during consultations. To ensure a representative selection, 4 experienced physicians from our lung cancer core team were interviewed. The questions covered general information about lung cancer, treatment options, radiotherapy, side effects, quality of life, preparations before treatment, and follow-up care. The questions were presented to GPT-4o (version as of July 2024) via OpenAI’s web interface, using the prompt: “I am a lung cancer patient looking for answers to the following questions.” Textbox 1 displays the questions and ChatGPT’s responses. The questions and answers were originally in German and translated for this publication. To ensure consistency, each question was posed to ChatGPT 5 times using the same prompt. Each question was presented individually using a standardized prompt structure, without engaging the model in an ongoing conversation or allowing follow-up questions or AI-generated additional prompts. This approach ensured that each response was independent and comparable across multiple trials. The responses were reviewed for variations in content and length by the primary researcher, and no substantial differences were identified.

Textbox 1. **Questions and responses from ChatGPT-4o.**

**Q1: What is lung cancer?**

ChatGPT: Lung carcinoma, also known as lung cancer, is a malignant neoplasm in the lung tissue. It develops when cells in the lungs grow uncontrollably and form tumors. There are 2 main types of lung cancer:

- Non-small cell lung carcinoma (NSCLC): This is the most common type and accounts for about 85% of cases. It includes various subtypes such as adenocarcinoma, squamous cell carcinoma, and large cell carcinoma.
- Small cell lung carcinoma (SCLC): This type is more aggressive and grows faster than non-small cell lung carcinoma. It is less common and accounts for about 15% of cases.

The main causes of lung cancer are tobacco smoking, inhalation of second-hand smoke, environmental and workplace exposure to certain pollutants, and genetic factors. Symptoms can include chronic cough, chest pain, weight loss, and shortness of breath. Diagnosis is usually made by imaging (eg, X-ray, CT scan) and biopsies. Treatment may include surgery, radiotherapy, chemotherapy, and targeted therapies, depending on the type and stage of the cancer.

**Q2: How can lung cancer be treated?**

ChatGPT: The treatment of lung cancer depends on the type (non-small cell or small cell) and the stage of the disease:

- Surgery: removal of the tumor and surrounding lung tissue, often for early stages of non-small cell lung cancer.
- Radiotherapy: High-energy radiation destroys cancer cells, which is useful for inoperable tumors or for follow-up treatment.
- Chemotherapy: Drug treatment to kill or inhibit the growth of cancer cells, used before or after surgery and in advanced stages.
- Targeted therapy: Drugs that attack specific molecular targets on cancer cells, particularly effective for certain genetic mutations.
- Immunotherapy: Stimulates the immune system to fight cancer cells, especially in advanced non-small cell lung cancer.
- Palliative therapy: Alleviates symptoms and improves quality of life in advanced cancer.

Treatment is often customized and may include a combination of these methods.

**Q3: What are the advantages of radiotherapy over other treatment options?**

ChatGPT: Radiotherapy offers several advantages in the treatment of lung cancer:

- Targeted treatment: precise radiation attacks the tumor directly, sparing the surrounding healthy tissue.
- Noninvasive: No surgical intervention is required, which is ideal for patients who cannot undergo surgery.
- Complementary to other therapies: Effective in combination with chemotherapy to improve outcomes.
- Applicability for inoperability: Useful for tumors that cannot be surgically removed.
- Pain relief: Relieves symptoms and improves quality of life in advanced cancer.
- Preservation of organ function: Preserves lung function better than complete removal of a lung section.
- Outpatient treatment: Often available as an outpatient therapy, minimizing hospitalization.

These benefits make radiotherapy a valuable treatment option for various stages of lung cancer.

**Q4: How is radiotherapy carried out for lung cancer?**

ChatGPT: Radiotherapy for lung cancer is carried out in several stages:

- Planning: Imaging techniques such as CT or MRI create detailed images of the tumor. A radiotherapist plans the exact dose and position of the radiation.
- Simulation: The patient is placed on a couch in the treatment position, often with special cushions or masks for stabilization. Markings on the skin or special tattoos help with precise alignment.
- Treatment: The actual radiotherapy takes place over several sessions (fractions), typically daily over several weeks. High-energy beams are directed at the tumor from different angles to achieve maximum effect and spare healthy tissue.
- Monitoring: regular reviews and imaging during the treatment phase to adjust therapy and monitor progress.

The treatment is painless and is performed on an outpatient basis, with each session lasting only a few minutes.

**Q5: What are the possible side effects of radiotherapy for lung cancer?**

ChatGPT: Radiotherapy for lung cancer can have several side effects, depending on the radiation dose and the area treated:

Acute side effects:

- Fatigue: frequent feeling of exhaustion.
- Skin reactions: Redness, irritation, or dryness in the irradiated area. Cough and breathing difficulties: Intensification of existing symptoms.

Late side effects:

- Scarring in the lungs can lead to long-term breathing problems. Pneumonia: Radiation pneumonitis is an inflammation of the lung tissue.
- Esophagitis: Inflammation and pain in the esophagus, making swallowing difficult.

Rare side effects:

- Heart problems: With radiation near the heart.
- Secondary tumors: Increased risk of other types of cancer in the irradiated area.

Side effects vary from person to person and can be mitigated by supportive measures and medication. Close monitoring by the treatment team helps to identify and treat side effects at an early stage.

**Q6: What precautions or preparations are necessary before the radiation treatment?**

ChatGPT: Several precautions and preparations are necessary before radiotherapy for lung cancer:

- Consultation and planning: a detailed consultation with the radiotherapist to discuss the treatment plan, possible side effects, and the expected course.
- Detailed imaging (CT and MRI) for precise localization of the tumor.
- Medical preparation: review and adjustment of current medications, as some may affect the radiation effect.
- Assessment of general health and lung function tests.
- Physical preparation: Making markings or tattoos on the skin for exact alignment during radiation.
- Possibly the creation of custom-made splints or masks to stabilize the treatment position.
- Nutrition and lifestyle advice: Advice on nutrition to strengthen the body.
- Advice to stop smoking and avoid alcohol to promote healing.
- Psychological support: access to counseling and support to manage anxiety and stress before treatment.

These preparations help to make the treatment more effective and safer.

**Q7: How does radiation affect the patient’s quality of life during and after treatment?**

ChatGPT: Radiotherapy can affect the patient’s quality of life in various ways, both during and after treatment:

During treatment:

- Fatigue: Frequent fatigue may limit daily activities.
- Skin reactions: Redness and irritation may cause discomfort.
- Breathing problems and coughing: Exacerbation of existing symptoms that interfere with everyday life.

After treatment:

- Recovery phase: Fatigue may last a few weeks, and return to normality often takes longer.
- Long-term effects: Scarring and radiation pneumonitis can cause breathing difficulties.
- Emotional distress: Fear of relapse and coping with physical changes.

Positive effects:

- Symptom relief: reduction of tumor pain and improvement in breathing can increase quality of life.
- Cancer control: Effective tumor control or reduction increases well-being and confidence.

Close cooperation with the medical team, supportive measures, and a healthy lifestyle help to improve quality of life during and after radiotherapy.

**Q8: What follow-up care is required after radiotherapy for lung cancer?**

ChatGPT: After radiotherapy for lung cancer, careful aftercare is crucial. This includes regular check-ups to monitor the response to treatment and detect possible relapses at an early stage. These usually include imaging such as CT or PET scans, blood tests, and lung function tests. It is also important to monitor and treat side effects of radiotherapy, such as pneumonia (radiation pneumonitis) or pulmonary fibrosis. Rehabilitation measures can help to improve lung function and increase quality of life. Psychosocial support is also an essential part of aftercare to provide emotional support for patients and their families.

### Evaluation of Readability

A readability analysis was conducted using the Modified Flesch Reading Ease (FRE) Formula for German. A well-established readability metric for the English language is the FRE scale [16]. The FRE measures the readability of a text in terms of its average sentence length (ASL) and the average number of syllables per word (ASW). It relies on the fact that short words or sentences are usually easier to understand than longer ones. For this analysis, we have used the modified FRE for the German language by Toni Amstad [17]: FRE (German)=180−ASL−(58.5×ASW).

Also, the 4th Vienna Formula (WSTF) was used. Unlike the FRE, the Vienna Formula (WSTF) has not been adapted for the German language. Instead, it is based on the work of Bamberger and Vanacek [18], who analyzed German textual material. They derived at least 5 versions of the Vienna Formula for prose and nonfiction texts. Typically, the fourth WSTF is used for text analysis. This metric is also based on average sentence length (ASL) and the proportion of words with three or more syllables (mean word syllables [MS]): WSTF=0.2656 × ASL +0.2744 × MS−1.6939.

The readability analysis and score calculation was performed using Python (version 3.8; Python Software Foundation) and its text processing libraries, such as nltk for sentence and word tokenization and a custom syllabification function for the German language. The FRE score was computed directly based on the modified formula for German. The WSTF score was calculated using the 4th Vienna Formula.

While the FRE and WSTF do not directly map to standard grade levels in the German language, readability categories were approximated to estimated educational levels to provide a practical interpretation of the required comprehension level. This approach allows for a more intuitive understanding of the readability of ChatGPT-generated responses in the context of patient education (see Table 1 for details).

**Table 1.** Interpretation of readability scores with estimated educational level: Modified Flesch Reading Ease (FRE) for German and 4th Vienna Formula (WSTF).

Description	FRE^a^	WSTF^b^	Estimated educational level (approximate)
Very difficult to read	0‐29	>14	University level
Difficult to read	30‐49	13‐14	Upper secondary (Grade 10-12/13)
Fairly difficult to read	50‐59	10‐13	Lower secondary (Grade 7‐10)
Average readability	60‐69	8‐10	Upper middle school (Grade 6)
Fairly easy to read	70‐79	7‐8	Lower middle school (Grade 5)
Easy to read	80‐89	5‐7	Upper elementary (Grade 4)
Very easy to read	90‐100	4‐5	Lower elementary (Grade 1‐3)

a FRE: Modified Flesch Reading Ease.

b WSTF: 4th Vienna Formula.

### Clinician Evaluation

Following the readability analysis, the 8 responses were independently evaluated by 6 clinicians experienced in lung cancer treatment, including 2 radiation oncologists, 2 medical oncologists, and 2 thoracic surgeons, all with 5‐12 years of experience working in specialized lung cancer centers with a university teaching function. This multidisciplinary approach ensured a comprehensive evaluation from different medical perspectives while remaining focused on lung cancer treatment. Clinicians received an information sheet outlining the study’s procedures and objectives. The question-answer pairs and evaluation sheet were provided in electronic form. Evaluators had no time limit to complete the assessment, scoring each response for relevance, correctness, and completeness using an ordinal 5-point Likert scale, with 1 indicating disagreement and 5 indicating complete agreement with the statements that the responses were relevant, correct, and complete, respectively. Respondents were also allowed to add additional comments to their evaluations.

### Patient Evaluation

**Figure 1.**
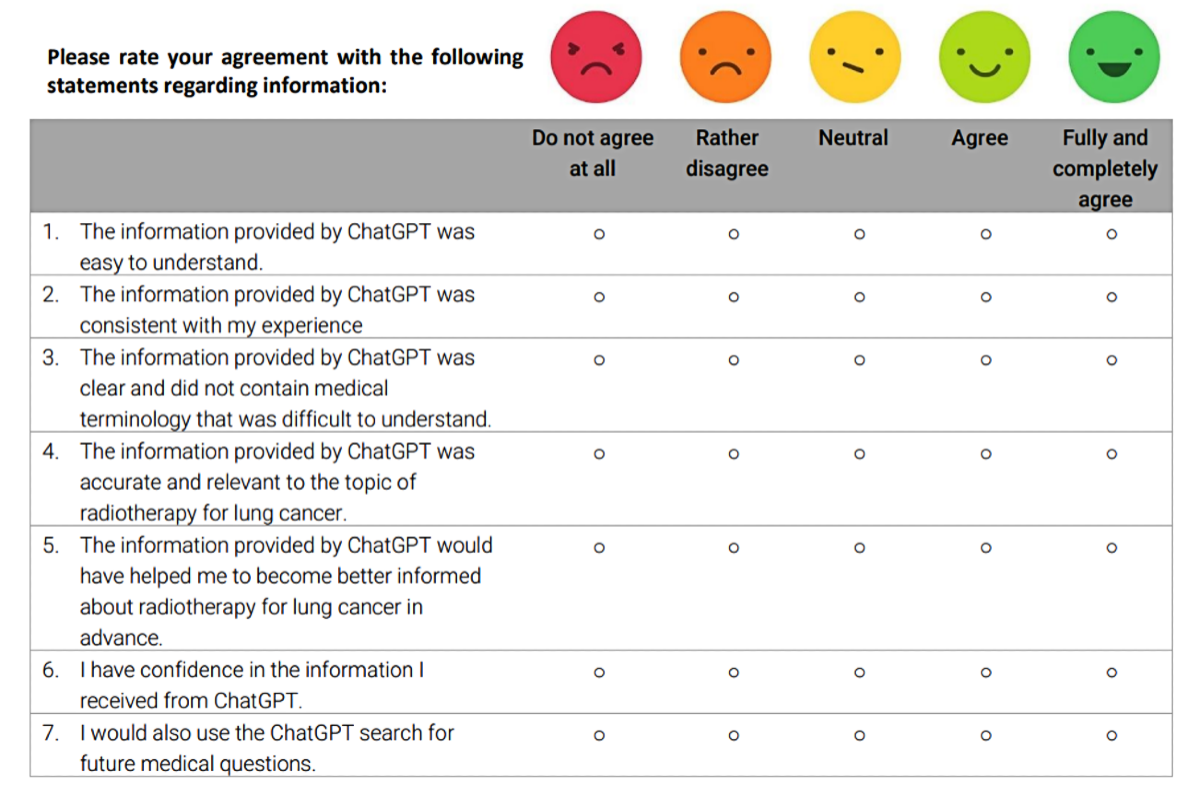
Example of a 5-point Likert scale presented to patients to rate ChatGPT’s responses.

Finally, the question-answer pairs were presented to patients with lung cancer during their first follow-up appointment after completing radiotherapy. Patients were invited to participate in a study evaluating an LLM for patient education. They received an information sheet outlining the study’s procedures and goals and were asked to sign a data security statement and provide informed consent before participation. After consenting, patients were given the question-answer pairs on paper sheets and had as much time as needed to complete the evaluation. The evaluation was based on 7 statements to assess ChatGPT’s performance in terms of comprehensibility, accuracy, relevance, and trustworthiness using a 5-point Likert scale (1=strongly disagree to 5=strongly agree; see Figure 1). In addition, they were asked whether the information made them feel better informed and if they would consider using ChatGPT for future medical questions. Patient responses from the paper forms were manually entered into Microsoft Office Excel (version 2410) by the primary researcher for further processing and analysis.

### Ethical Considerations

The local Ethics Committee of Ludwig-Maximilians-University Munich (LMU) approved the study protocol in August 2023 (approval 23‐0742). The study was conducted in accordance with the Declaration of Helsinki, and all patients provided signed written consent to participate. To ensure privacy and confidentiality, all collected data were anonymized before analysis and no personal identifiable information was stored or shared. Data were handled in compliance with institutional and national data protection regulations. Participants did not receive any financial or material compensation for their participation in the study.

### Statistical Analysis

Data are reported using descriptive statistics, including median, mean, and SD. Statistical analyses were performed using Microsoft Office Excel (version 2410). Figures were generated using Python (version 3.8) with the Matplotlib library. Data extracted from tables was structured in Pandas DataFrames for analysis and plotting.

## Results

### Evaluation of Readability

Table 1 shows the interpretation of readability scores with an estimated educational level. The FRE scores ranged from 6.3 to 42.3, with a mean of 23.4 (SD 11.2), classifying most responses as “very difficult to read” (University level). Similarly, the WSTF scores ranged from 10.6 to 16.8, with a mean of 13.8 (SD 2.1). Most responses were in the “very difficult to read” category, with some being “difficult” (upper secondary, grade 10-12/13) or “fairly difficult” (lower secondary, grade 7‐10; see Table 2).

**Table 2.** Readability analysis of ChatGPT’s responses to questions 1-8: Modified Flesch Reading Ease (FRE) and 4th Vienna Formula (WSTF), displaying individual scores of answers 1-8, mean (SD), and minimum-maximum.

Answer	FRE^a^	FRE Interpretation	WSTF^b^	WSTF Interpretation
A1	42.3	Difficult to read	10.6	Fairly difficult to read
A2	12.6	Very difficult to read	16.8	Very difficult to read
A3	23.9	Very difficult to read	14.4	Very difficult to read
A4	35.8	Difficult to read	10.8	Fairly difficult to read
A5	21.1	Very difficult to read	13.6	Difficult to read
A6	28.2	Very difficult to read	14.1	Very difficult to read
A7	16.6	Very difficult to read	14.7	Very difficult to read
A8	6.3	Very difficult to read	15.	Very difficult to read
Minimum-maximum	6.3‐42.3	—^c^	10.6‐16.8	—
Mean (SD)	23.4 (11.2)	—	13.8 (2.1)	—

a FRE: Modified Flesch Reading Ease.

b WSTF: 4th Vienna Formula.

c Not available.

### Clinician Evaluation

Table 3 presents the evaluation of ChatGPT’s responses by 6 clinicians experienced in treating lung cancer: 2 radiation oncologists, 2 medical oncologists, and 2 thoracic surgeons.

The mean scores for relevance ranged from 3.7, SD 0.94 (responses 2 [treatment] and 3 [advantages of radiotherapy]) to 4.3, SD 0.75 (response 8 [follow-up]). Correctness scores varied between 3.5, SD 0.50 (response 7 [quality of life]) and 4.3, SD 0.75 (response 8 [follow-up]). Completeness ratings ranged from 3.5, SD 0.50 (responses 2 [treatment], 5 [side effects], and 7 [quality of life]) to 4.2, SD 0.69 (response 8 [follow up]). Overall, responses showed variability in performance, with relevance and correctness achieving higher mean scores than completeness. Notably, response 8 (follow-up) scored the highest across all 3 dimensions (relevance: 4.3, SD 0.75; correctness: 4.3, SD 0.75; and completeness: 4.2, SD 0.69), while response 7 (quality of life) scored the lowest for correctness (3.5, SD 0.50).

A thoracic surgeon commented that ChatGPT did not discuss chances of treatment success and recurrence rates. A medical oncologist commented that the role of multidisciplinary tumor boards should have been mentioned. A radiation oncologist commented that there was no differentiation between radiotherapy modalities.

**Table 3.** Clinician ratings of ChatGPT’s responses (1–8) for relevance, correctness, and completeness. Scores are based on a 5-point Likert scale, where 1 represents the lowest and 5 represents the highest score.

Response to questions	Mean (SD)	Ratings on Likert scale, n (%)				
		1	2	3	4	5
Response 1						
Relevance	3.8 (1.07)	0 (0)	1 (17)	1 (17)	2 (33)	2 (33)
Correctness	4.2 (0.37)	0 (0)	0 (0)	0 (0)	5 (83)	1 (17)
Completeness	3.5 (0.76)	0 (0)	1 (17)	1 (17)	4 (67)	0 (0)
Response 2						
Relevance	3.7 (0.94)	0 (0)	1 (17)	1 (17)	3 (50)	1 (17)
Correctness	3.7 (0.75)	0 (0)	0 (0)	3 (50)	2 (33)	1 (17)
Completeness	3.5 (0.50)	0 (0)	0 (0)	3 (50)	3 (50)	0 (0)
Response 3						
Relevance	3.7 (0.94)	0 (0)	1 (17)	1 (17)	3 (50)	1 (17)
Correctness	3.7 (0.75)	0 (0)	0 (0)	3 (50)	2 (33)	1 (17)
Completeness	3.7 (0.47)	0 (0)	0 (0)	2 (33)	4 (67)	0 (0)
Response 4						
Relevance	4.3 (0.47)	0 (0)	0 (0)	0 (0)	4 (67)	2 (33)
Correctness	3.8 (0.37)	0 (0)	0 (0)	1 (17)	5 (83)	0 (0)
Completeness	3.8 (0.37)	0 (0)	0 (0)	1 (17)	5 (83)	0 (0)
Response 5						
Relevance	4.2 (0.90)	0 (0)	0 (0)	2 (33)	1 (17)	3 (50)
Correctness	4.0 (0.58)	0 (0)	0 (0)	1 (17)	4 (67)	1 (17)
Completeness	3,5 (0.50)	0 (0)	0 (0)	3 (50)	3 (50)	0 (0)
Response 6						
Relevance	3.8 (0.90)	0 (0)	0 (0)	3 (50)	1 (17)	2 (33)
Correctness	4.0 (0.00)	0 (0)	0 (0)	0 (0)	6 (100)	0 (0)
Completeness	4.0 (0.00)	0 (0)	0 (0)	0 (0)	6 (100)	0 (0)
Response 7						
Relevance	4.0 (0.82)	0 (0)	0 (0)	2 (33)	2 (33)	2 (33)
Correctness	3.5 (0.50)	0 (0)	0 (0)	3 (50)	3 (50)	0 (0)
Completeness	3.5 (0.50)	0 (0)	0 (0)	3 (50)	3 (50)	0 (0)
Response 8						
Relevance	4.3 (0.75)	0 (0)	0 (0)	1 (17)	2 (33)	3 (50)
Correctness	4.3 (0.75)	0 (0)	0 (0)	1 (17)	2 (33)	3 (50)
Completeness	4.2 (0.69)	0 (0)	0 (0)	1 (17)	3 (50)	2 (33)

### Patient Evaluation

The responses generated by ChatGPT were evaluated by 30 consecutive patients who underwent radiation therapy for lung cancer between June 2024 and October 2024 at the University Hospital LMU Munich during their first follow-up examination 6 weeks after treatment completion. The median age of the 19 male and 11 female patients was 66 years (48‐87 years). A total of 26 of those patients had non-small cell lung cancer (NSCLC), while 4 patients had small cell lung cancer (SCLC). In addition, 12 patients received concomitant chemotherapy, and 10 patients received stereotactic body radiotherapy (SBRT). A total of 5 patients were treated using magnetic resonance-guided radiotherapy (MRgRT).

Results of the patient evaluation are summarized in Table 4. The highest-rated statement was “The information provided by ChatGPT was easy to understand,” with a mean score of 4.4 (SD 0.61), where 94% of patients rated it as “agree” or “strongly agree.” Similarly, the statement “The information provided by ChatGPT was accurate and relevant to radiotherapy for lung cancer” received a high mean score of 4.2 (SD 0.83), with 87% of patients rating it positively. The statement “The information provided by ChatGPT was consistent with my experience” achieved a mean score of 4.1 (SD 0.63), reflecting alignment with patient expectations. Similarly, the statement “The information provided by ChatGPT was clear and did not contain medical terminology that was difficult to understand” received a mean score of 4.1 (SD 0.81), with 80% of patients giving positive feedback. This highlights ChatGPT’s strength in delivering accessible and jargon-free information.

In contrast, statements related to usability and trustworthiness received slightly lower ratings. “The information provided by ChatGPT would have helped me to become better informed about radiotherapy for lung cancer in advance” and “I would also use ChatGPT for future medical questions” both scored a mean of 3.9 (SD 0.94). In addition, the statement “I have confidence in the information I received from ChatGPT” scored 4.0 (SD 0.84).

**Table 4.** Patient’s ratings of statements 1‐7. Scores are based on a 5-point Likert scale, where 1 represents the lowest and 5 the highest score (1=strongly disagree, 5=strongly agree).

Statement		Mean (SD)	Ratings on likert scale, n (%)				
			1	2	3	4	5
The information provided by ChatGPT was easy to understand.		4.4 (0.61)	0 (0)	0 (0)	2 (7)	14 (47)	14 (47)
The information provided by ChatGPT was consistent with my experience.		4.1 (0.63)	0 (0)	0 (0)	5 (17)	18 (60)	7 (23)
The information provided by ChatGPT was clear and did not contain medical terminology that was difficult to understand.		4.1 (0.81)	0 (0)	1 (3)	5 (17)	13 (43)	11 (37)
The information provided by ChatGPT was accurate and relevant to the topic of radiotherapy for lung cancer.		4.2 (0.83)	0 (0)	2 (7)	2 (7)	14 (47)	12 (40)
The information provided by ChatGPT would have helped me to become better informed about radiotherapy for lung cancer in advance.		3.9 (0.94)	0 (0)	3 (10)	6 (20)	12 (40)	9 (30)
I have confidence in the information I received from ChatGPT.		4.0 (0.84)	0 (0)	1 (3)	8 (27)	12 (40)	9 (30)
I would also use the ChatGPT search for future medical questions.		3.9 (0.94)	0 (0)	3 (10)	6 (20)	12 (40)	9 (30)

## Discussion

### Principal Findings

Providing accessible and understandable information is a key component of patient-centered care, particularly in oncology. Research has shown that patients with cancer often seek information from sources other than their health care providers, with the internet serving as a primary resource [19]. However, existing online resources frequently fail to address patients’ specific questions, especially in radiation oncology and often exceed recommended complexity levels [20,21]. Against this background, our study explores the potential of the most widely used and broadly adopted LLM, ChatGPT [22], making it a relevant and practical model for evaluating real-world applications in patient communication and education in lung cancer radiotherapy.

This study evaluated the benefits and risks of using ChatGPT to educate patients undergoing radiotherapy for lung cancer. The analysis included a multifaceted evaluation of ChatGPT-generated content, including readability assessment, clinician evaluation, and patient feedback. The main findings indicate that while ChatGPT’s responses are often technically complex and rated as “difficult to read” based on objective readability measures (FRE and WSTF), patients still perceived the information as clear and understandable. Clinicians rated the responses positively for relevance and correctness but noted some limitations in completeness.

### Comparison With Previous Work

The readability analysis of ChatGPT’s responses revealed that the FRE and WSTF scores classified most responses as “very difficult to read” or “difficult to read,” which may limit accessibility, particularly for individuals with lower health literacy. The low readability scores are primarily due to the extensive use of complex medical terminology and long sentence structures, which increase the calculated ASL and ASW values, thereby reducing readability. While the FRE and WSTF scores do not have direct grade-level equivalents in German, texts classified as “very difficult to read” or “difficult to read,” typically require upper secondary education or higher for full comprehension. In addition, the complexity of the prompt can influence the readability of responses, as more detailed inquiries tend to generate longer, more technical answers, which may further reduce readability. These findings are consistent with previous studies in other medical domains, which have similarly reported low readability scores for AI-generated content, suggesting that readability challenges are a common limitation across various medical specialties and not specific to lung cancer [23-26]. Furthermore, lung cancer education inherently involves complex terminology, multidisciplinary treatment approaches, and a broad spectrum of disease presentations, all of which may contribute to lower readability scores compared to simpler medical topics. These findings align with studies indicating that cancer-related information on the internet is generally not well-tailored to patients’ needs [27]. Despite this, the patient evaluation showed that ChatGPT’s responses were perceived as easy to understand (mean score 4.4, SD 0.61), possibly because the survey was conducted post-therapy when patients were already familiar with relevant topics and terminology. One possible strategy to improve readability in patient education materials is the fine-tuning of LLMs with curated, patient-friendly datasets or the integration of real-time readability adjustments that simplify sentence structure while maintaining medical accuracy. In addition, a hybrid approach involving AI-generated content reviewed by clinicians may enhance accessibility without compromising correctness.

The clinician evaluation of ChatGPT highlighted its strengths in relevance and correctness but noted limitations in completeness. Response 8, for example, performed best across all dimensions (relevance: 4.3, SD 0.75; correctness: 4.3, SD 0.75; and completeness: 4.2, SD 0.69), while Response 7 demonstrated inconsistencies, scoring the lowest for correctness (3.5, SD 0.50). These findings align with other studies assessing ChatGPT’s accuracy in answering questions about lung cancer [28,29] and other queries in radiotherapy [30,31]. Interestingly, another study found that ChatGPT achieved high qualitative ratings for factual accuracy, conciseness, and completeness, closely mirroring expert responses [32]. The lower completeness scores suggest that ChatGPT responses, while relevant and mostly accurate, may omit critical clinical details. This limitation could be mitigated by refining prompting strategies to ensure more detailed outputs or integrating clinician oversight in AI-assisted patient education.

Patients rated ChatGPT highly for clarity and relevance, but usability and trust received comparatively lower scores. Statements like “I would also use ChatGPT for future medical questions” (3.9, SD 0.94) and “I have confidence in the information I received from ChatGPT” (4.0, SD 0.84) highlight areas where trust and reliability could be improved. Lower trustworthiness and usability scores suggest that while patients find ChatGPT-generated responses clear and relevant, concerns remain regarding the credibility of AI-generated medical information. Future implementations could improve trust through clinician oversight, AI transparency measures, and integration with evidence-based sources.

### Considerations for Clinical Integration

LLMs like ChatGPT are often approached cautiously in health care due to concerns about trust, security, privacy, and ethics [33,34]. While ChatGPT is sometimes criticized for lacking a human touch and empathy [35], studies have found its responses to be more empathetic than those of clinicians in specific scenarios [36], especially for sensitive health topics where patients may feel uncomfortable consulting clinicians, nonsentient chatbot tools may offer valuable support [32].

Despite ongoing concerns about “hallucinations,” where LLMs generate plausible but incorrect answers [10,11], no potential harm was identified in ChatGPT’s responses in this study. OpenAI, the developer of ChatGPT, acknowledges the possibility of inaccurate outputs, likely contributing to health care providers’ reluctance to adopt LLM chatbots for patient communication and education. However, other studies have shown that ChatGPT can provide highly accurate and complete responses comparable to virtual patient-clinician communication in radiation oncology [32]. To minimize the risk of misinformation, future AI-driven patient education tools should incorporate source attribution, real-time fact-checking, and clinician oversight. In addition, models specifically trained on verified medical datasets may help reduce the occurrence of incorrect or misleading information.

### Strengths and Limitations

First, a key strength of this study is its comprehensive evaluation approach, combining readability metrics with both clinician and patient assessments.

This study has several limitations. First, the questions were formulated by the study team based on input from clinicians experienced in lung cancer treatment, rather than being directly collected from patients. While this approach ensured clinical relevance and reflected frequently encountered consultation topics, it may have limited the diversity of clinical scenarios and the representativeness of the findings from a broader patient perspective. Second, the study was conducted in German, which could affect the generalizability of results, as ChatGPT’s performance may vary across languages [37,38]. Third, this study used GPT-4, released by OpenAI in May 2024, a paid subscription model with superior accuracy and coherence compared to the free GPT-3.5 version, which may limit accessibility by the general population. Fourth, another key limitation of this study is the lack of standardized criteria for assessing AI-generated medical responses. Future research should focus on developing structured evaluation frameworks, integrating expert consensus and establishing domain-specific benchmarks to ensure consistent assessment of AI-generated content.

Fifth and finally, while our study focused on ChatGPT, the most popular and earliest publicly released conversational LLM [22], other models, such as Bard (Google), LLAMA (Meta), and Claude (Anthropic), show promise in addressing oncology queries. However, no comparative analysis of these alternative models was conducted in this study, as the primary objective was to assess the feasibility and quality of ChatGPT’s responses as a widely used reference model in patient education. ChatGPT was selected due to its widespread adoption and superior response quality demonstrated in previous studies compared to other LLMs [24,39,40]. Previous research has highlighted differences among LLMs in terms of response accuracy, completeness, and readability in health care applications, including radiotherapy [39-44]. Future studies should explore how various LLMs perform specifically in the context of patient education in lung cancer, to identify the most suitable tools for clinical integration.

### Conclusion and Future Directions

In conclusion, ChatGPT demonstrates significant potential as a supplementary tool for patient education in radiation oncology, particularly for patients undergoing radiotherapy for lung cancer. Its ability to provide clear and relevant information highlights its value in enhancing patient understanding and engagement in their treatment journey. However, limitations in completeness, accuracy, and trust underscore the importance of careful review and supplementation by health care professionals.

Further development of AI tools should focus on improving readability through fine-tuning on patient-friendly datasets or integrating real-time readability adaptation, while maintaining medical accuracy. Incorporating clinician oversight into AI-generated content could enhance both reliability and trust. In addition, the development of standardized evaluation frameworks for AI-generated health information will be essential to ensure consistent quality assessment. With continued research and refinement, ChatGPT and similar technologies have the potential to revolutionize patient education and support health care providers in delivering accurate, accessible, and personalized care.

## Abbreviations

AI: artificial intelligence
ASL: average sentence length
ASW: average number of syllables per word
FRE: Flesch Reading Ease
LLM: large language model
LMU: Ludwig-Maximilians-University Munich
MRgRT: magnetic resonance-guided radiotherapy
NLP: natural language processing
NSCLC: non-small cell lung cancer
RI: Readability Index
SBRT: stereotactic body radiotherapy
SCLC: small cell lung cancer
WSTF: 4th Vienna Formula
